# Evolution of In Vitro Antimicrobial Susceptibility of Equine Clinical Isolates in France between 2016 and 2019

**DOI:** 10.3390/ani10050812

**Published:** 2020-05-07

**Authors:** Albertine Léon, Sophie Castagnet, Karine Maillard, Romain Paillot, Jean-Christophe Giard

**Affiliations:** 1 LABÉO Frank Duncombe, 14053 CAEN, France; sophie.castagnet@laboratoire-labeo.fr (S.C.); karine.maillard@laboratoire-labeo.fr (K.M.); romain.paillot@laboratoire-labeo.fr (R.P.); 2 Normandie Univ, UNICAEN, U2RM, 14033 Caen, France; jean-christophe.giard@unicaen.fr; 3 Normandie Univ, UNICAEN, Biotargen, 14033 Caen, France

**Keywords:** antibiotic resistance, horse pathogens, epidemiology

## Abstract

**Simple Summary:**

The emergence and the spread of antimicrobial drug resistant bacteria around the world is a major public health issue. In fact, the transmission of these bacteria from animals to humans has been already observed. In this context, the close relationships between horses and humans may contribute to cross-infection. Our objective in this study was to describe the antimicrobial susceptibility profiles of major equine pathogens over a 4-year period (2016–2019). For this purpose, more than 7800 bacterial isolates collected from horses in France with different types of infection were phenotypically analysed for their antimicrobial susceptibility. An increase in the resistance of *Staphylococcus aureus* and *Enterobacter* spp. was observed, especially between 2016 and 2019, with the percentage of multi-drug resistant strains rising from 24.5% to 37.4%, and from 26.3% to 51.7%, respectively. Our results point to the need to support veterinary antimicrobial stewardship to encourage the proper use of antibiotics.

**Abstract:**

The present study described the evolution of antimicrobial resistance in equine pathogens isolated from 2016 to 2019. A collection of 7806 bacterial isolates were analysed for their in vitro antimicrobial susceptibility using the disk diffusion method. The most frequently isolated pathogens were group C *Streptococci* (27.0%), *Escherichia coli* (18.0%), *Staphylococcus aureus* (6.2%), *Pseudomonas aeruginosa* (3.4%), *Klebsiella pneumoniae* (2.3%) and *Enterobacter* spp. (2.1%). The majority of these pathogens were isolated from the genital tract (45.1%, n = 3522). With the implementation of two French national plans (named ECOANTIBIO 1 and 2) in 2012–2016 and 2017–2021, respectively, and a reduction in animal exposure to veterinary antibiotics, our study showed decreases in the resistance of group C *Streptococci*, *Klebsiella pneumoniae* and *Escherichia coli* against five classes, four classes and one class of antimicrobials tested, respectively. However, *Staphylococcus aureus*, *Escherichia coli* and *Enterobacter* spp. presented an increased resistance against all the tested classes, excepted for two fifths of *E. coli.* Moreover, the percentages of multi-drug resistant strains of *Staphylococcus aureus* and *Enterobacter* spp. also increased from 24.5% to 37.4% and from 26.3% to 51.7%, respectively. The data reported here are relevant to equine practitioners and will help to improve knowledge related to antimicrobial resistance in common equine pathogens.

## 1. Introduction

Since the beginning of the 21st century, the emergence of multi-drug-resistant bacteria has become a major public health concern and a priority for all international institutions such as the World Health Organization (WHO), the Food and Agriculture Organization of the United Nations (FAO) and the World Organization for Animal Health (or Organisation Internationale des Epizooties-OIE), which provide guidelines to mitigate the development of resistant bacteria [1,2,3]. 

In France, two governmental programmes were initiated over the 2012–2016 (ECOANTIBIO 1) and 2017–2021 (ECOANTIBIO 2) periods to reduce the veterinary use of antibiotics and to preserve the therapeutic arsenal for serious illness cases [4,5]. The objectives of the first programme were both quantitative (reduce, by 25%, the exposure of animals to antibiotics over a 5-year period) and qualitative (a reduction in the use of critical antibiotics in veterinary medicine including fluoroquinolones and last-generation cephalosporins) in order to reduce the occurrence of antimicrobial resistance, which is an international concern in terms of human and animal health [4]. 

The second programme focuses on incentivisation rather than regulatory measures by promoting communication, training, the use of alternatives to antibiotics, improvements in preventive measures for infectious diseases and the provision of the best tools for diagnosis and monitoring antibiotic sales and resistance [5].

In this context, several international studies have described the prevalence of resistant bacteria in equine samples in South Africa [6], Canada [7,8], Switzerland [9] and the United Kingdom [10]. Some of them reported a high level of resistance in horse’s bacteria: from 26.6% to 50% of multi-drug resistant (MDR) strains [6,10], 60% and 68% of isolates were phenotypically extended-spectrum beta-lactamase (ESBL)-producing and methicillin-resistant, respectively [9]. 

In France, retrospective studies concerning data collected from 2006 to 2016 have been recently published and demonstrated a potential role of equids as a reservoir [11,12], and this report aims to evaluate the situation and its progression with the analysis of more than 7000 samples collected between 2016 and 2019.

## 2. Materials and Methods 

From January 2016 to December 2019, bacterial isolates collected from horses (with suspicion of bacterial infection and prior antimicrobial treatment) by numerous practitioners in France were included in the study. Data for 2016 came from our previous manuscript [11]. Because samples came from several farms over very large areas, they could not be considered as geographically clustered. Analyses were performed in the Veterinary Microbiology diagnostics unit of the LABÉO Research and Diagnostic Institute. Strains were isolated on agar media (Columbia agar with 5% sheep blood or Columbia CNA agar with 5% sheep blood and eosin methylene blue agar). Strains were identified by Gram staining and commercially available identification systems, such as the API and VITEK 2 Compact^®^ systems (bioMérieux, mArcy l’Etoile, France) or since 2018 (April), MALDI-TOF mass spectrometry (Microflex; Bruker Daltonics, Bremen, Germany), according to the manufacturers’ instructions. 

Antimicrobial susceptibility testing (AST) was performed using the disc diffusion method on Mueller–Hinton agar (enriched with 5% sheep blood for *Streptococcus* spp.) according to the recommendations of the CA-SFM/EUCAST (Comité de l’antibiogramme de la Société Française de Microbiologie/The European Committee on Antimicrobial Susceptibility Testing) [13]. The categorisations of antimicrobial susceptibility testing were carried out using the CA-SFM recommendations for antimicrobial drugs only used in veterinary medicine (as Ceftiofur, Cefquinome, Flumequine, Enrofloxacine and Marbofloxacine). For other drugs also used in human medicine, EUCAST recommendations were taken into account. After 18–24 h of incubation at 37 °C, the diameters of growth inhibition around the discs were measured using SIRSCAN (I2A, Montpellier, France) and interpreted to show bacteria were susceptible, intermediate or resistant according to CA-SFM/EUCAST clinical breakpoints. Bacteria that were categorised as “intermediate” were subsequently considered as “resistant” in our study. Bacterial isolates were evaluated for their susceptibilities to β-lactams, polymyxins, aminoglycosides, tetracycline, macrolides, rifampicin, sulphonamides and fluoroquinolones. Due to their intrinsic resistance to low levels of aminoglycosides, high concentration (HC) aminoglycosides discs were used against *Streptococci*.

Statistical analysis was performed using the XLStat software. The chi-square test was used to test for significant changes in antimicrobial resistance among each bacterial species between one year and its previous year. The temporal trends in the prevalence of antimicrobial resistance were investigated for each antimicrobial compound using the Cochran Armitage trend test. For these analyses, *p* < 0.05 were considered as significant.

## 3. Results

### 3.1. Identification and Distribution of Bacterial Isolates

**Figure 1:**
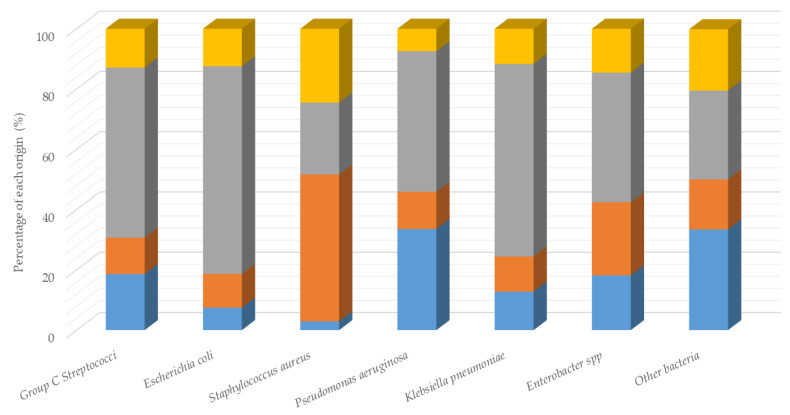
Repartition of sampling origins (%)—in blue, respiratory; in orange, cutaneous; in grey, genital; and in yellow, others—according to pathogen type.

In a 4-year period, 7806 bacterial isolates were included (2016: n = 1895; 2017: n =1978; 2018: n = 2125; 2019: n = 1808). These isolates were clustered from genital (45.1%; n = 3522), respiratory (22.1%; n = 1728) and cutaneous (16.3%; n = 1273) origins and other origins such as digestive or ophthalmic (16.4%; n = 1283). The most frequently isolated pathogens were group C *Streptococci* including *Streptococcus equi* subsp. *zooepidemicus*, *Streptococcus equi* subsp. *equi* and *Streptococcus dysgalactiae* subsp. *equisimilis* (27.0%, n = 2118); *Escherichia coli* (18.0%, n = 1382); *Staphylococcus aureus* (6.2%, n = 482); *Pseudomonas aeruginosa* (3.4%, n = 268); *Klebsiella pneumoniae* (2.3%, n = 180); and *Enterobacter* spp (2.1%, n = 165). The relationship between pathogen types and sampling origins (i.e., types of infection) is illustrated in Figure 1.

### 3.2. Antimicrobial Susceptibility

#### 3.2.1. GRAM Positive Bacteria

Group C *Streptococci* were the most frequent isolated bacteria (27.0%), mainly from genital samples (56.4%) (Figure 1). No resistance was observed against penicillins and cephalosporins (Table 1). Between 2016 and 2019, the frequencies of Group C *Streptococci* highly resistant to streptomycin HC and kanamycin HC have significantly decreased over time, from 5.5% to 0.5% (*p* < 0.0001) and from 5.3% to no resistant strains (*p* < 0.0001), respectively. Despite an increased resistance to macrolides, rifampicin and sulphonamides observed in 2017 compared to 2016, the level of resistance decreased significantly in subsequent years. Concerning tetracycline, more than 82% of streptococcal isolates were resistant in 2016 and 2017, with a significant reduction to near 72% in 2018 and 60% in 2019 (Table 1). 

Most of these tetracycline-resistant group C *Streptococci* isolates were also of genital origin. They represented 83.5% in 2016, 90.4% in 2017, 82.1% in 2018 and 64.8% in 2019 (Figure S1a). 

*Staphylococcus aureus (S. aureus)* is mainly isolated from cutaneous (48.8%) and genital samples (23.9%) (Figure 1). As shown in Table 2, a significant overall increase in the resistance to all antimicrobial drugs—except penicillin, amoxicillin, cefoxitin, streptomycin and erythromycin—used against *S. aureus* was observed, especially for aminoglycosides and tetracycline for which the percentage of resistant strains was more than 40% in 2019. The level of *S. aureus* resistant to oxacillin and cefoxitin (used as markers for methicillin resistance and extended to other β-lactams) increased from 15.8% to 28% over time.

#### 3.2.2. GRAM Negative Bacteria

*Escherichia coli (E. coli)* was the second most frequent bacterium isolated (18%), mainly from genital samples (68.9%). Resistance to penicillins varied significantly during the 2016–2019 period, decreasing between 2016 and 2018, from 39.5% to 27.4%, for amoxicillin and from 31.4% to 18.3% for amoxicillin combined with clavulanic acid, and then increasing between 2018 and 2019, to 32.8% and 19.4%, respectively. Less than 6.2% of *E. coli* strains were resistant to cephalosporins (C3G and C4G) and quinolones. Concerning the resistance to streptomycin, after a significant decrease from 33.1% in 2016 to 26.2% in 2017 (*p* = 0.048), the highest percentage of resistant bacteria was measured in 2019, at 43.4% (*p* < 0.0001). For other aminoglycosides (such as kanamycin and gentamicin), resistant *E. coli* strains represented less than 11.2%. More than 76% and 67% of *E. coli* strains remained susceptible and sensitive to tetracycline and sulphonamides, respectively, and the percentage was 94.6% for quinolones (Table 3).

The majority of resistant *E. coli* strains have been isolated from genital samples , where resistance to streptomycin increased from 28.4% in 2016 to 36.7% in 2019 (Figure S1c). 

*Pseudomonas aeruginosa* (*P. aeruginosa*) represents 3.4% of the total bacteria and was mainly isolated from genital (46.6%) and respiratory (33.6%) samples (Figure 1). The analysis was limited to cefquinome (C4G), gentamicin and marbofloxacin, which are the only antimicrobials clinically relevant for *P. aeruginosa* in veterinary medicine. The frequency of strains resistant to these three agents was less than 15% during the 2016–2019 period (Table 4).

From 2018 to 2019, the frequency of strains resistant to cefquinome and gentamicin decreased, from 28.6% to 13.3% and from 32.1% to 20.0%, respectively, in respiratory samples (Figure S1d). Concerning resistant *P. aeruginosa* strains isolated from genital samples, a decrease was observed for cefquinome (from 26.1% in 2017 to 12.5% in 2019) and for gentamicin (from 13% in 2017 to 7.5% in 2019). These percentages have been calculated from the data in Table S1, and the variations are represented in terms of the numbers of isolates in Figure S1d.

*Klebsiella pneumoniae (K. pneumoniae)* represented 2.3% of all the bacteria and were mainly isolated from genital samples (63.9%) (Figure 1). The number of isolated strains doubled in four years from 30 to 60 strains (Table 5). A large increase in the resistance level to all antimicrobial agents was observed in 2017, especially for amoxicillin–clavulanic acid (from 12.9% to 42.4%), streptomycin (from 29.0% to 48.5%), tetracycline (from 25.8% to 48.5%) and sulphonamides (from 32.3% to 51.5%). Decreases were then measured in 2018 and confirmed in 2019, except for cephalosporins (change from 5.4% to 10%). 

Genital samples contained most of the resistant strains, with 63.9%. After an important increase in 2017, with 66.7% of genital strains resistant against tetracycline, 58.3% against amoxicillin with clavulanic acid and 50% against streptomycin and sulphonamides, the levels of strains resistant against all antimicrobial agents were below 10% in 2019 (Figure S1e).

*Enterobacter* spp represented 2.1% of total bacteria and was mainly isolated from genital samples (43.0%) (Figure 1). Only the evolution of strains resistant to streptomycin and kanamycin was significant over the 4-year period (*p* = 0.022 and *p* = 0.044 respectively). The percentage of *Enterobacter* spp. strains resistant to streptomycin increased significantly from 23.7% in 2016 to 50.0% in 2017 (*p* = 0.025) and to 55.2% in 2019. Similar evolution was observed for kanamycin (*p* = 0.024) and gentamicin (*p* = 0.008) resistance from 18.4% in 2016 to more than 41% in 2017. The frequency of strains resistant to cephalosporins varied between 15.8% and 34.6% for ceftiofur and between 10.3% and 21.7% for cefquinome. Strains resistant to tetracycline and sulphonamides increased from 21.1% to 37.9% and 48.3%, respectively. Concerning quinolone resistance, 27.6% of isolated strains were resistant to flumequine, 10.3% to enrofloxacin and 6.9% to marbofloxacine in 2019 (Table 6).

The distribution of resistant strains according to sample origins revealed that genital samples contained the largest number of resistant bacteria (43%). In 2019, 21.4 % of *Enterobacter* spp. isolated from the genital tract were resistant to cefquinome and tetracycline, 42.9 % to streptomycin, 35.7% to kanamycin, and 28.6% to gentamicin and sulphonamides (Figure S1f). 

### 3.3. Multi-Drug Resistant (MDR) Bacteria

Table 7 shows the overall level and trend of MDR bacteria (defined as non-susceptible to at least three different classes of antibiotic usually efficient) according to bacterial species. Because few antimicrobial compounds have been tested against *P. aeruginosa*, this species was excluded from the analysis. For group C *Streptococci*, the level of MDR increased from 2016 (10.7%) to 2017 (18.9%) before decreasing to 0.5% in 2019. For *S. aureus*, the percentage of MDR increased from 24.5% in 2016 to 37.4% in 2019. For *E. coli*, the level of MDR remained similar during the 2016–2019 period (average of resistance was 22.0%). For *K. pneumoniae*, the level of MDR, after an increase from 38.7% in 2016 to 51.5% in 2017, decreased to 11.7% in 2019. Finally, for *Enterobacter* spp., this level doubled from 26.3% in 2016 to 51.7% in 2019.

## 4. Discussion

Horses are now recognised to be potential reservoirs of antimicrobial resistance, which can be transmitted to other animal and human pathogens [11,12,14,15,16]. Equine pathogens with zoonotic potential should be carefully taken into account, especially *Enterobacteriaceae* producing extended-spectrum beta-lactamases (ESBL), *P. aeruginosa* or methicillin-resistant *Staphylococcus aureus* (MRSA), as described by the WHO [17]. 

Human infection with Group C *Streptococci* is not frequent but can lead to severe diseases, such as septicemia, meningitis or arthritis [18,19,20]. In horses, *Streptococcus equi* subsp *equi* and *Streptococcus equi* subsp *zooepidemicus* (*Streptococcus zooepidemicus*) are the most important bacterial pathogens encountered, the causative agent of Strangles and the leading cause of bacterial infection, respectively [21,22,23]. Only very few (less than 1%) strains resistant to penicillins and cephalosporins were identified in the 4-year study, in agreement with other studies conducted in France [11,12] and throughout the world [6,7,8,9,10]. However, a significantly increased resistance to tetracycline, macrolides, rifampicin and sulphonamides was measured in 2017, followed by a significant decrease in 2018 and 2019. These results were well correlated with the trend of MDR *Streptococci* that represented 0.5% in 2019. *Streptococcus zooepidemicus* is frequently associated with uterine infections but can also induce persistent subclinical infection of the mare due to the presence of “dormant” bacterial colonies in the endometrium, with an impact on fertility. This stage of dormancy is often associated with an increased resistance to penicillin (not linked to the acquisition of a resistance gene but due to the absence of replication) [24]. The instillation of a bacterial growth medium (bActivate) in the uterus has been shown to “reactivate” dormant *Streptococcus zooepidemicus*, which subsequently allows the efficient use of antibiotics to clear the persistent infection [24]. It is also important to note that *Streptococcus zooepidemicus* is often associated with secondary bacterial infections in horses after respiratory virus infections [25]. The use of vaccination against the primary pathogen should also be considered as an indirect way to reduce antibiotic use through the prevention of secondary bacterial infections, as previously demonstrated for equine influenza vaccinations [26]. 

Concerning *S. aureus,* an increased frequency of MRSA strains was observed between 2016 and 2019. As shown in Table 2, the level of oxacillin-resistant strains (used as a methicillin resistance marker) increased by more than 10% (from 15.8% in 2016 to 27.1% in 2019). More strains resistant against aminoglycosides and tetracyclines were also isolated. In parallel, we showed that the level of MDR *S. aureus* also increased during the same period. As the level of MRSA remained stable between 2013 and 2016, the current result raises questions regarding long-term vigilance and the correct use of antimicrobial compounds [11]. Moreover, in a previous study, we have demonstrated the predominance of ST398 MRSA isolates since 2011. Since this ST398 type is known to cause outbreaks in horses and to colonise/infect humans, hygiene measures and appropriate antimicrobial use should be maintained and reinforced in order to limit the transmission of *S. aureus* between horses as well as between horses and humans [27]. 

Concerning *E. coli*, resistance to cephalosporins and quinolones represented, in 2019, less than 10% of all isolated strains, as observed in previous years [11,12], which is well correlated with their limited use in the field because of their critical categorisation by authorities. Indeed, one study that measured the trends in the antimicrobial susceptibility of several pathogens, such as *Enterobacteriacae*, isolated between 1979 and 2010 from foals with sepsis highlighted a decrease in ceftiofur activity [28]. For other drugs, the resistance levels measured in this study were similar to the levels reported before 2016, with the exception of those for streptomycin, which reached 43.4% in 2019. Even if it was significantly reduced two-fold compared to 2009 and 2010, where 94.8% and 90.4% were resistant, respectively, this percentage remained high between 2016 and 2019 (26.2% in 2017 and 43.4% in 2019). This may be explained by the fact that *E. coli* strains were mainly isolated from the genital tract and that streptomycin represents the main treatment for such infections, thereby promoting the selection of streptomycin (STR)-resistant *E. coli* isolates. This again points to the necessity of performing antimicrobial susceptibility testing before any antimicrobial treatment. 

In a lower proportion than *E. coli*, two other Enterobacteria were also analysed, *K. pneumoniae* (2.3% of isolated bacteria) and *Enterobacter spp* (2.1% of isolated bacteria). For *K. pneumoniae*, unexpectedly, the frequency of strains resistant against all antimicrobial agents increased in 2017 before reaching levels of resistance similar to those observed during 2006–2016 period. For *Enterobacter* spp., the same observation was made in 2017, with a subsequent maintenance of high levels of resistance against aminoglycosides, streptomycin and kanamycin especially, until 2019. The MDR proportion of *Enterobacter* spp. also doubled between 2016 and 2019. As mentioned above, the increased resistance of *K. pneumoniae* and *Enterobacter* spp. observed in 2017 could be linked to the transition between the two French national programmes leading to reduced vigilance regarding the antimicrobials used. However, although *Enterobacter* spp. represented the smallest sample of pathogens in this study (2.1%), attention must be paid to multi-drug resistant strains whose levels remained very high until 2019. Marbofloxacine appeared to be the compound against which few *Enterobacter* were resistant, and it should be used with caution to avoid a therapeutic impasse. 

As described in literature [9,11,29], only a few antimicrobial agents such as cefquinome, gentamicin and marbofloxacine remained active against *P. aeruginosa*. Moderate levels of resistance (around 8.6%, 14.7% and 4.7% for cefquinome, gentamicin and marbofloxacine, respectively) were measured between 2016 and 2019, as already observed in 2015 [11]. *Pseudomonas aeruginosa* strains were mainly isolated from the respiratory and genital tracts, where antimicrobial drugs are more targeted because of reproduction and horse racing, inducing less selective pressure. 

Overall, the antimicrobial resistance of the bacteria studied here increased in 2017, which was the pivotal year between the two governmental programmes implemented in France to counteract antimicrobial resistance. A decrease was subsequently observed. Nevertheless, high levels of MDR persist as of 2019, especially in more than 37% of *S. aureus* and 51% of *Enterobacter* spp. This situation will have to be carefully monitored in the future. The use of these antimicrobials has to be moderated in order to prevent the spread of resistance.

The majority of the bacterial collection analysed in this study was isolated from the genital tract, which is linked to the primary diagnostic activity of the LABÉO Veterinary Microbiology unit. Consequently, this study may lack representativeness regarding other compartments.To date, the use of antimicrobials in equine veterinary medicine remains a necessity in many cases because the methods for the prevention of bacterial infections in horses are limited, with few or no vaccines available. 

In this context, antimicrobials are essential to equine health, and this study underlines the importance of performing antimicrobial susceptibility testing in order to optimise antimicrobial therapy in horses and to reduce the occurrence of resistance. Such studies are essential for evaluating the evolution of antimicrobial resistance and its potential threat to public health. In addition, such data are key indicators for the impact of national or international plans leading to a reduction in the spread of multi-drug resistant bacteria and may help in drawing up new guidelines. The analysis of the resistance mechanisms displayed by these major bacteria is warranted. The use of whole genome sequencing of strains of interest will prove to be invaluable for investigating molecular epidemiology. 

## 5. Conclusions

During the 2016-2019 period, decreases in the resistance of group C Streptococci and Klebsiella pneumoniae against at least four classes of antimicrobials, were observed. In addition, Staphylococcus aureus, and Enterobacter spp. presented an increased resistance against all the classes tested. In this context, the percentages of multi-drug resistant strains of these species increased from 24.5% to 37.4% and from 26.3% to 51.7%, respectively. For E. coli, the situation was mixed with a decrease against penicillins and an increase against streptomycin and sulphonamides. Our study indicates that horses may be considerate as reservoirs of antimicrobial resistant pathogens and underlines the importance of performing antimicrobial susceptibility testing in order to optimize antimicrobial therapy in horses and to reduce the occurrence of these resistances.

## Figures and Tables

**Table 1:** Percentage of resistant group C *Streptoccoci* isolates per year.

Antibiotic Category		Year	2016	2017	2018	2019
		(Number of Strains)	(692)	(598)	(454)	(374)
Penicillins		PEN	0.1	0.3	0	0
		AMX ** (*p* = 0.016)	0.7	0.2	0	0
		OXA	0.1	0.3	0	0
		AMC ** (*p* = 0.011)	0.7	0.0 *	0	0
				(*p* = 0.037)		
Cephalosporins	3rd	CEF ** (*p* = 0.049)	0.4	0	0	0
	4th	CEQ	0.1	0	0	0
Aminoglycosides		STR ^HC^ ** (*p* < 0.0001)	5.5	3.8	0.0 *	0.5

		KAN ^HC^ ** (*p* < 0.0001)	5.3	4.5	0.2 *	0
					(*p* < 0.0001)	
		GEN^HC^	0.6	1.2	0.2	0
Tetracycline		TET ** (*p* < 0.0001)	82.1	87.0 *	71.6 *	58.6 *
				(*p* < 0.0001)	(*p* < 0.0001)	(*p* < 0.0001)
Macrolides		ERY ** (*p* < 0.0001)	11.1	22.1*	10.3 *	2.1 *
				(*p* < 0.0001)	(*p* < 0.0001)	(*p* < 0.0001)
Rifampicin		RIF	15.5	47.8 *	22.0 *	16.6 *
				(*p* < 0.0001)	(*p* < 0.0001)	(*p* = 0.049)
Sulphonamides		SXT ** (*p* < 0.0001)	4.8	15.6 *	0.7 *	0
				(*p* < 0.0001)	(*p* < 0.0001)	

PEN—penicillin; AMX—amoxicillin; AMC—amoxicillin-clavulanic acid; CEF—ceftiofur; CEQ—cefquinome; STR—streptomycin; KAN—kanamycin; GEN—gentamicin TET—tetracycline; ERY—erythromycin; RIF—rifampicin; SXT—trimethoprim-sulfamethoxazole; OXA—oxacillin, marker of methicillin resistance; * Chi-square test compared to the previous year, *p* < 0.05; ** Cochran–Armitage trend test, *p* < 0.05; ^HC^—High concentration. The percentage of resistance is categorised by cell colours: green for resistance ≤10%, yellow for resistance between 10% and 30%, pink for resistance between 30% and 50%, and red for resistance >50%.

**Table 2:** Percentage of resistant *Staphylococcus aureus* per year.

Antibiotic Category		Year	2016	2017	2018	2019
		(Number of Strains)	(139)	(118)	(118)	(107)
Penicillins		PEN	43.9	47.5	47.5	56.1
		AMX	43.2	46.6	47.5	55.1
		OXA ** (*p* = 0.045)	15.8	22.9	22.0	27.1
		AMC ** (*p* = 0.021)	17.3	22.9	22.6	28.0
Cephalosporins	2nd	FOX	17.3	22.9	22.6	28.0
	3rd	CEF ** (*p* = 0.045)	17.3	22.9	22.6	28.0
	4th	CEQ ** (*p* = 0.031)	17.3	22.9	22.6	28.0
Aminoglycosides		STR	20.9	11.0 *	11.0	17.8
				(*p* = 0.033)		
		KAN ** (*p* = 0.003)	23.0	31.4	32.2	41.1
		GEN ** (*p* = 0.001)	21.6	30.5	32.2	41.1
Tetracycline		TET ** (*p* = 0.01)	27.3	35.6	35.6	43.9
Macrolides		ERY	5.8	5.1	4.2	8.4
Rifampicin		RIF ** (*p* < 0.001)	2.9	11.0 *	15.3	16.8
				(*p* = 0.009)		
Sulphonamides		SXT ** (*p* = 0.008)	6.5	3.4	12.7 *	14.0
					(*p* = 0.008)	
Fluoroquinolones		ENO ** (*p* = 0.002)	1.4	2.5	5.9	9.3
		MAR ** (*p* = 0.002)	1.4	1.7	5.9	9.3

PEN—penicillin; AMX—amoxicillin; AMC—amoxicillin-clavulanic acid; CEF—ceftiofur; CEQ—cefquinome; STR—streptomycin; KAN—kanamycin; GEN—gentamicin; TET—tetracycline; ERY—erythromycin; RIF—rifampicin; SXT—trimethoprim-sulfamethoxazole; ENO—enrofloxacin; MAR—marbofloxacine; OXA—oxacillin, marker of methicillin resistance; FOX—cefoxitin, marker of methicillin resistance; * Chi-square test compared to the previous year, *p* < 0.05; ** Cochran–Armitage trend test, *p* < 0.05. The percentage of resistance is categorised by cell colours: green for resistance ≤10%, yellow for resistance between 10% and 30%, pink for resistance between 30% and 50%, and red for resistance >50%.

**Table 3:** Percentage of resistant *Escherichia coli* per year.

Antibiotic Category		Year	2016	2017	2018	2019
		(Number of Strains)	(344)	(325)	(372)	(341)
Penicillins		AMX ** (*p* = 0.02)	39.5	33.2	27.4	32.8
		AMC ** (*p* < 0.0001)	31.4	21.2 *	18.3	19.4
				(*p* = 0.003)		
Cephalosporins	3rd	CEF	6.1	5.8	6.2	3.5
	4th	CEQ	5.8	5.8	6.2	3.8
Aminoglycosides		STR ** (*p* = 0.005)	33.1	26.2*	28.2	43.4 *
				(*p* = 0.048)		(*p* < 0.0001)
		KAN	9.0	8.9	9.1	11.1
		GEN	6.1	7.1	8.9	6.7
Tetracycline		TET	20.6	21.2	23.1	22.6
Sulphonamides		SXT	31.4	28.3	28.8	32.6
Quinolones/Fluoroquinolones		NAL	4.9	3.4	5.4	3.2
		FLU	4.9	3.4	5.4	3.2
		ENO	3.2	3.4	2.4	3.2
		MAR	2.9	3.4	2.4	2.9

AMX—amoxicillin; AMC—amoxicillin-clavulanic acid; CEF—ceftiofur; CEQ—cefquinome; STR—streptomycin; KAN—kanamycin; GEN—gentamicin; TET—tetracycline; SXT—trimethoprim-sulfamethoxazole; ENO—enrofloxacin; MAR—marbofloxacine; FLU—flumequine; NAL—nalidixic acid, marker of fluoroquinolone resistance. * Chi-square test compared to the previous year, *p* < 0.05; ** Cochran–Armitage trend test, *p* < 0.05. The percentage of resistance is categorised by cell colours: green for resistance ≤10%, yellow for resistance between 10% and 30%, and pink for resistance between 30% and 50%.

**Table 4:** Percentage of resistant *Pseudomonas aeruginosa* per year.

Antibiotic Category		Year	2016	2017	2018	2019
		(Number of Strains)	(59)	(70)	(75)	(64)
Cephalosporin	4th	CEQ	11.9	14.3	14.7	12.5
Aminoglycosides		GEN	10.2	8.6	14.7	10.9
Fluoroquinolones		MAR	1.7	0.0	0.0	4.7

CEQ—cefquinome; GEN—gentamicin; MAR—marbofloxacine. * Chi-square test compared to the previous year, *p* < 0.05; ** Cochran–Armitage trend test, *p* < 0.05. The percentage of resistance is categorised by cell colours: green for resistance ≤10% and yellow for resistance between 10% and 30%.

**Table 5:** Percentage of resistant *Klebsiella pneumoniae* per year.

Antibiotic Category		Year	2016	2017	2018	2019
		(Number of Strains)	(31)	(33)	(56)	(60)
Penicillins		AMC	12.9	42.4 *	16.1 *	10.0
				(*p* = 0.009)	(*p* = 0.006)	
Cephalosporins	3rd	CEF	9.7	21.2	5.4 *	10.0
					(*p* = 0.022)	
	4th	CEQ	9.7	21.2	5.4 *	10.0
					(*p* = 0.022)	
Aminoglycosides		STR ** (*p* = 0.008)	29.0	48.5	25.0 *	13.3
					(*p* = 0.024)	
		KAN	3.2	12.1	7.1	6.7
		GEN	6.5	21.2	7.1	6.7
Tetracycline		TET ** (*p* = 0.017)	25.8	48.5	25.0 *	13.3
					(*p* = 0.024)	
Sulphonamides		SXT ** (*p*=0.006)	32.3	51.5	26.8 *	15.0
					(*p* = 0.019)	
Quinolones/Fluoroquinolones		NAL ** (*p* = 0.049)	19.4	21.2	8.9	8.3
		FLU	12.9	21.2	8.9	8.3
		ENO	9.7	18.2	3.6 *	5.0
					(*p* = 0.02)	
		MAR	3.2	9.1 *	1.8	3.3
				(*p* = 0.02)		

AMC—amoxicillin-clavulanic acid; CEF—ceftiofur; CEQ—cefquinome; STR—streptomycin; KAN—kanamycin; GEN—gentamicin; TET—tetracycline; SXT—-trimethoprim-sulfamethoxazole; ENO—enrofloxacin; MAR—marbofloxacine; FLU—flumequine; NAL—nalidixic acid, marker of fluoroquinolone resistance. * Chi-square test compared to the previous year, *p* < 0.05; ** Cochran–Armitage trend test, *p* < 0.05. The percentage of resistance is categorised by cell colours: green for resistance ≤10%, yellow for resistance between 10% and 30%, pink for resistance between 30% and 50%, and red for resistance >50%.

**Table 6:** Percentage of resistant *Enterobacter* spp. per year.

Antibiotic Category		Year	2016	2017	2018	2019
		(Number of Strains)	(38)	(46)	(52)	(29)
Cephalosporins	3rd	CEF	15.8	30.4	34.6	27.6
	4th	CEQ	13.2	21.7	21.2	10.3
Aminoglycosides		STR ** (*p* = 0.022)	23.7	50.0 *	44.2	55.2
				(*p* = 0.025)		
		KAN ** (*p* = 0.044)	18.4	41.3 *	36.5	44.
				(*p* = 0.024)		8
		GEN	18.4	45.7 *	42.3	41.4
				(*p* = 0.008)		
Tetracycline		TET	21.1	32.6	36.5	37.9
Sulphonamides		SXT	21.1	45.7	42.3	48.3
Quinolones/Fluoroquinolones		NAL	21.1	17.4	23.1	27.6
		FLU	21.1	17.4	23.1	27.6
		ENO	7.9	8.7	13.5	10.3
		MAR	2.6	4.3	7.7	6.9

CEF—ceftiofur; CEQ—cefquinome; STR—streptomycin; KAN—kanamycin; GEN—gentamicin; TET—tetracycline; SXT—trimethoprim-sulfamethoxazole; ENO—enrofloxacin; MAR—marbofloxacine; FLU—flumequine; NAL—nalidixic acid, marker of fluoroquinolone resistance. * Chi-square test compared to the previous year, *p* < 0.05; ** Cochran–Armitage trend test, *p* < 0.05. The percentage of resistance is categorised by cell colours: green for resistance ≤10%, yellow for resistance between 10% and 30%, pink for resistance between 30% and 50%, and red for resistance >50%.

**Table 7:** Percentage of bacteria resistant to three or more antimicrobial classes.

	*Streptococcus* (Group C) ** (*p* < 0.001)	Staphylococcus aureus ** (*p* = 0.029)	*E. coli*	Klebsiella pneumoniae ** (*p* = 0.001)	*Enterobacter* spp. ** (*p* = 0.048)
2016	10.7	24.5	22.7	38.7	26.3
2017	18.9	31.4	21.2	51.5	45.6
2018	3.1	33.1	21.8	26.8	44.2
2019	0.5	37.4	22.6	11.7	51.7

** Cochran–Armitage trend test, *p* < 0.05.

## Supplementary Materials

The following are available online at https://www.mdpi.com/2076-2615/10/5/812/s1, Table S1: Number of resistant strains of the major bacterial species isolated over a 4-years period (2016–2019) according to the origin. R-respiratory; D-digestive; C-cutaneous; G-genital and O-other, Figure S1: Total (blue) and resistant (red) relevant Gram-positive (a, b) and Gram-negative (c, d, e, f) bacteria isolated per year and major sample groups.
