# Supplementary material for: Evolution of In Vitro Antimicrobial Susceptibility of Equine Clinical Isolates in France between 2016 and 2019
Source: Animals (Basel). 2020 May 7;10(5):812. doi: 10.3390/ani10050812 (PMC7278474; doi:10.3390/ani10050812)
Supplement: Supplementary file 1 [file animals-10-00812-s001.zip › Figure S1_animals_78119.pptx]

## Slide 1
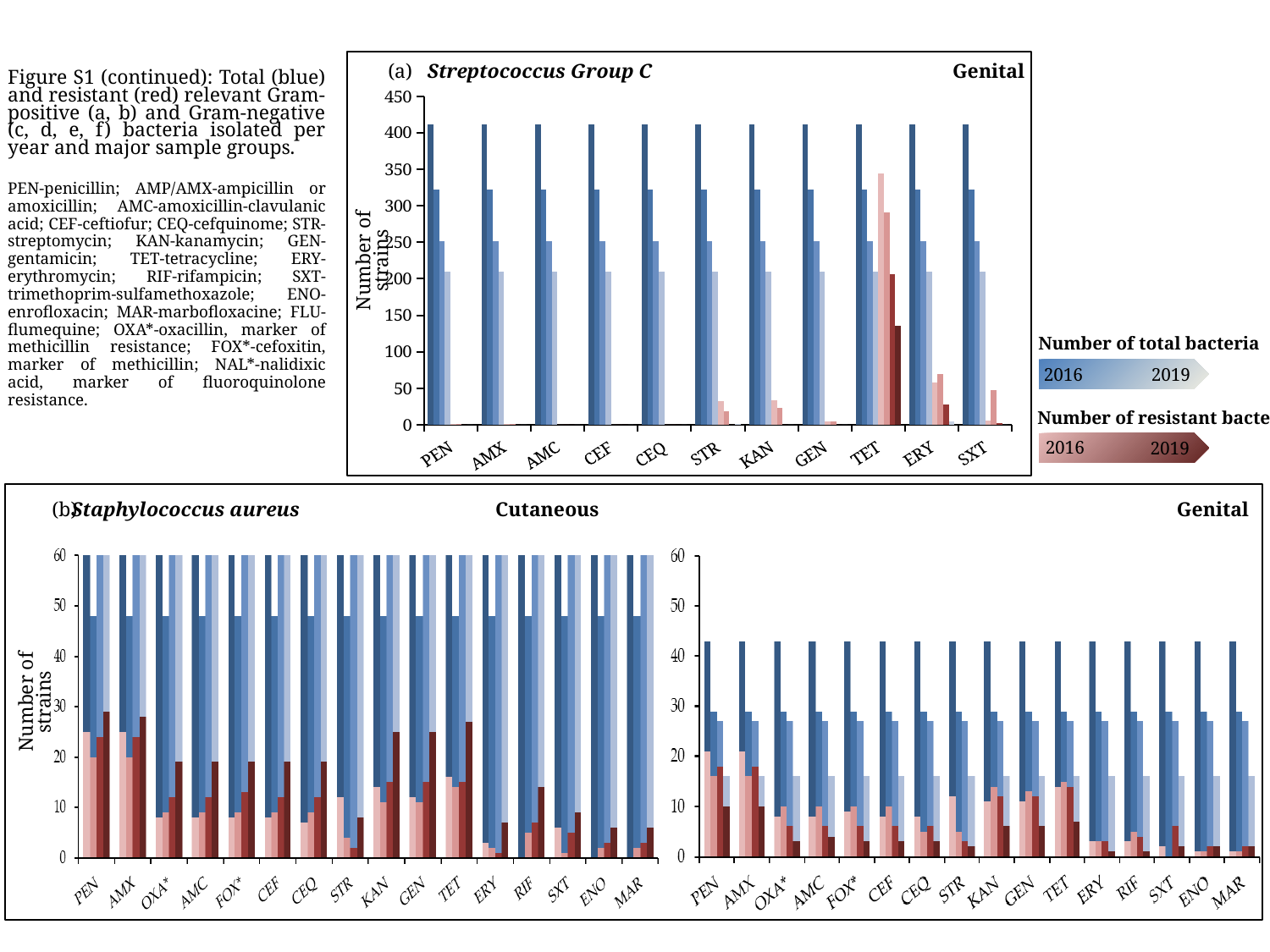

(a)
Streptococcus Group C
Genital
Number of total bacteria
2016
2019
Number of resistant bacteria
2016
2019
(b)
Staphylococcus aureus
Cutaneous
Genital
### Chart
| Category | total 2016 | total 2017 | total 2018 | total 2009 | res | res | res | res |
|---|---|---|---|---|---|---|---|---|
| PEN | 412.0 | 322.0 | 251.0 | 210.0 | 1.0 | 1.0 | 0.0 | 0.0 |
| AMX | 412.0 | 322.0 | 251.0 | 210.0 | 1.0 | 1.0 | 0.0 | 0.0 |
| AMC | 412.0 | 322.0 | 251.0 | 210.0 | 0.0 | 0.0 | 0.0 | 0.0 |
| CEF | 412.0 | 322.0 | 251.0 | 210.0 | 0.0 | 0.0 | 0.0 | 0.0 |
| CEQ | 412.0 | 322.0 | 251.0 | 210.0 | 0.0 | 0.0 | 0.0 | 0.0 |
| STR | 412.0 | 322.0 | 251.0 | 210.0 | 32.0 | 19.0 | 0.0 | 1.0 |
| KAN | 412.0 | 322.0 | 251.0 | 210.0 | 33.0 | 23.0 | 0.0 | 0.0 |
| GEN | 412.0 | 322.0 | 251.0 | 210.0 | 4.0 | 4.0 | 0.0 | 0.0 |
| TET | 412.0 | 322.0 | 251.0 | 210.0 | 344.0 | 291.0 | 206.0 | 136.0 |
| ERY | 412.0 | 322.0 | 251.0 | 210.0 | 58.0 | 70.0 | 28.0 | 4.0 |
| SXT | 412.0 | 322.0 | 251.0 | 210.0 | 6.0 | 47.0 | 2.0 | 0.0 |
Figure S1 (continued): Total (blue) and resistant (red) relevant Gram-positive (a, b) and Gram-negative (c, d, e, f) bacteria isolated per year and major sample groups.
PEN-penicillin; AMP/AMX-ampicillin or amoxicillin; AMC-amoxicillin-clavulanic acid; CEF-ceftiofur; CEQ-cefquinome; STR-streptomycin; KAN-kanamycin; GEN-gentamicin; TET-tetracycline; ERY-erythromycin; RIF-rifampicin; SXT-trimethoprim-sulfamethoxazole; ENO-enrofloxacin; MAR-marbofloxacine; FLU-flumequine; OXA*-oxacillin, marker of methicillin resistance; FOX*-cefoxitin, marker of methicillin; NAL*-nalidixic acid, marker of fluoroquinolone resistance.
Number of strains
Number of strains

## Slide 2
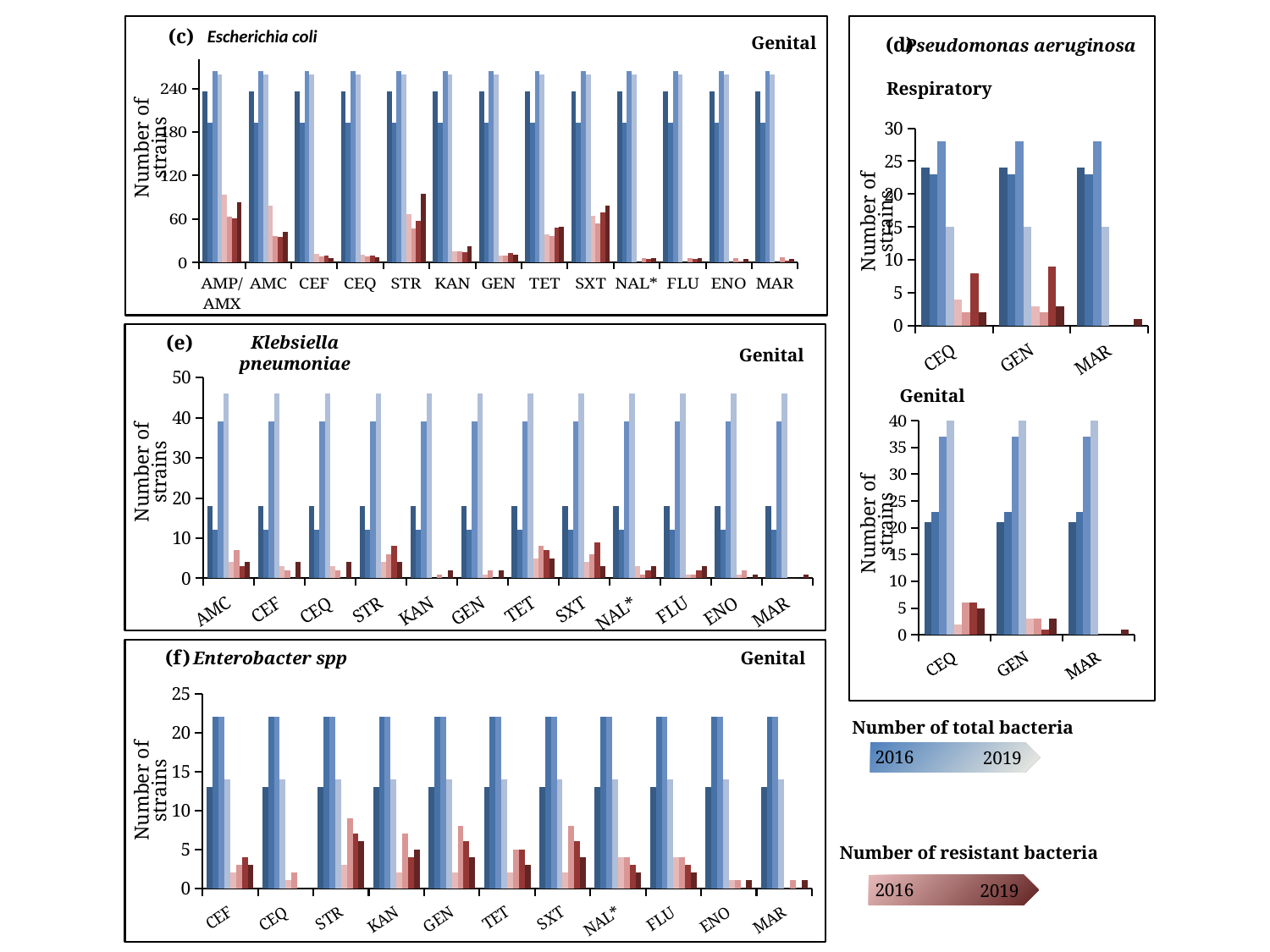

(d)
Pseudomonas aeruginosa
Respiratory
### Chart
| Category | 2016 | 2017 | 2018 | 2019 | 2016 | 2017 | 2018 | 2019 |
|---|---|---|---|---|---|---|---|---|
| CEQ | 24.0 | 23.0 | 28.0 | 15.0 | 4.0 | 2.0 | 8.0 | 2.0 |
| GEN | 24.0 | 23.0 | 28.0 | 15.0 | 3.0 | 2.0 | 9.0 | 3.0 |
| MAR | 24.0 | 23.0 | 28.0 | 15.0 | 0.0 | 0.0 | 0.0 | 1.0 |Genital
### Chart
| Category | 2016 | 2017 | 2018 | 2019 | 2016 | 2017 | 2018 | 2019 |
|---|---|---|---|---|---|---|---|---|
| CEQ | 21.0 | 23.0 | 37.0 | 40.0 | 2.0 | 6.0 | 6.0 | 5.0 |
| GEN | 21.0 | 23.0 | 37.0 | 40.0 | 3.0 | 3.0 | 1.0 | 3.0 |
| MAR | 21.0 | 23.0 | 37.0 | 40.0 | 0.0 | 0.0 | 0.0 | 1.0 |Number of total bacteria
2016
2019
Number of resistant bacteria
2016
2019
(c)
Escherichia coli
Genital
### Chart
| Category | 2016 | 2017 | 2018 | 2019 | 2016 | 2017 | 2018 | 2019 |
|---|---|---|---|---|---|---|---|---|
| AMP/AMX | 236.0 | 193.0 | 264.0 | 259.0 | 94.0 | 64.0 | 61.0 | 83.0 |
| AMC | 236.0 | 193.0 | 264.0 | 259.0 | 79.0 | 37.0 | 36.0 | 43.0 |
| CEF | 236.0 | 193.0 | 264.0 | 259.0 | 12.0 | 9.0 | 10.0 | 6.0 |
| CEQ | 236.0 | 193.0 | 264.0 | 259.0 | 11.0 | 9.0 | 10.0 | 7.0 |
| STR | 236.0 | 193.0 | 264.0 | 259.0 | 67.0 | 47.0 | 58.0 | 95.0 |
| KAN | 236.0 | 193.0 | 264.0 | 259.0 | 16.0 | 16.0 | 14.0 | 22.0 |
| GEN | 236.0 | 193.0 | 264.0 | 259.0 | 10.0 | 10.0 | 13.0 | 11.0 |
| TET | 236.0 | 193.0 | 264.0 | 259.0 | 39.0 | 37.0 | 48.0 | 49.0 |
| SXT | 236.0 | 193.0 | 264.0 | 259.0 | 65.0 | 54.0 | 69.0 | 78.0 |
| NAL* | 236.0 | 193.0 | 264.0 | 259.0 | 0.0 | 6.0 | 5.0 | 6.0 |
| FLU | 236.0 | 193.0 | 264.0 | 259.0 | 0.0 | 6.0 | 5.0 | 6.0 |
| ENO | 236.0 | 193.0 | 264.0 | 259.0 | 0.0 | 6.0 | 2.0 | 5.0 |
| MAR | 236.0 | 193.0 | 264.0 | 259.0 | 0.0 | 7.0 | 3.0 | 5.0 |
(e)
Klebsiella pneumoniae
Genital
### Chart
| Category | 2016 | 2017 | 2018 | 2019 | 2016 | 2017 | 2018 | 2019 |
|---|---|---|---|---|---|---|---|---|
| AMC | 18.0 | 12.0 | 39.0 | 46.0 | 4.0 | 7.0 | 3.0 | 4.0 |
| CEF | 18.0 | 12.0 | 39.0 | 46.0 | 3.0 | 2.0 | 0.0 | 4.0 |
| CEQ | 18.0 | 12.0 | 39.0 | 46.0 | 3.0 | 2.0 | 0.0 | 4.0 |
| STR | 18.0 | 12.0 | 39.0 | 46.0 | 4.0 | 6.0 | 8.0 | 4.0 |
| KAN | 18.0 | 12.0 | 39.0 | 46.0 | 0.0 | 1.0 | 0.0 | 2.0 |
| GEN | 18.0 | 12.0 | 39.0 | 46.0 | 1.0 | 2.0 | 0.0 | 2.0 |
| TET | 18.0 | 12.0 | 39.0 | 46.0 | 5.0 | 8.0 | 7.0 | 5.0 |
| SXT | 18.0 | 12.0 | 39.0 | 46.0 | 4.0 | 6.0 | 9.0 | 3.0 |
| NAL* | 18.0 | 12.0 | 39.0 | 46.0 | 3.0 | 1.0 | 2.0 | 3.0 |
| FLU | 18.0 | 12.0 | 39.0 | 46.0 | 1.0 | 1.0 | 2.0 | 3.0 |
| ENO | 18.0 | 12.0 | 39.0 | 46.0 | 1.0 | 2.0 | 0.0 | 1.0 |
| MAR | 18.0 | 12.0 | 39.0 | 46.0 | 0.0 | 0.0 | 0.0 | 1.0 |(f)
Enterobacter spp
Genital
### Chart
| Category | 2016 | 2017 | 2018 | 2019 | 2016 | 2017 | 2018 | 2019 |
|---|---|---|---|---|---|---|---|---|
| CEF | 13.0 | 22.0 | 22.0 | 14.0 | 2.0 | 3.0 | 4.0 | 3.0 |
| CEQ | 13.0 | 22.0 | 22.0 | 14.0 | 1.0 | 2.0 | 0.0 | 0.0 |
| STR | 13.0 | 22.0 | 22.0 | 14.0 | 3.0 | 9.0 | 7.0 | 6.0 |
| KAN | 13.0 | 22.0 | 22.0 | 14.0 | 2.0 | 7.0 | 4.0 | 5.0 |
| GEN | 13.0 | 22.0 | 22.0 | 14.0 | 2.0 | 8.0 | 6.0 | 4.0 |
| TET | 13.0 | 22.0 | 22.0 | 14.0 | 2.0 | 5.0 | 5.0 | 3.0 |
| SXT | 13.0 | 22.0 | 22.0 | 14.0 | 2.0 | 8.0 | 6.0 | 4.0 |
| NAL* | 13.0 | 22.0 | 22.0 | 14.0 | 4.0 | 4.0 | 3.0 | 2.0 |
| FLU | 13.0 | 22.0 | 22.0 | 14.0 | 4.0 | 4.0 | 3.0 | 2.0 |
| ENO | 13.0 | 22.0 | 22.0 | 14.0 | 1.0 | 1.0 | 0.0 | 1.0 |
| MAR | 13.0 | 22.0 | 22.0 | 14.0 | 0.0 | 1.0 | 0.0 | 1.0 |Number of strains
Number of strains
Number of strains
Number of strains
Number of strains
